# Supplementary material for: A null model of the mouse whole-neocortex micro-connectome
Source: Nat Commun. 2019 Aug 29;10:3903. doi: 10.1038/s41467-019-11630-x (PMC6715727; doi:10.1038/s41467-019-11630-x)
Supplement: Supplementary file 1 — Supplementary Information [file 41467_2019_11630_MOESM1_ESM.pdf]

Supplementary Information for Reimann et al.:

"A null model of the mouse whole-neocortex micro-connectome"

1 Supplementary Notes

1.1 Incomplete mapping

To model the mapping between brain regions from the voxelized connectivity data, we had to make a strong assumption on the nature of said mapping: That it is continuous with only linear scaling. We also assumed that the mapping tends to cover most of the source region (see above), although the model will account for it if the data strongly indicates so. For example, feedback projections in the somatomotor system tended to cover the source regions only incompletely (Supplementary Figure 3 a, columns "data"). Note, how SSp-ll was only targeted by projections from neurons in MOp associated with red, and from neurons in SSs associated with red or green. Conversely, SSp-m were targeted by the regions of MOp and SSs associated with green or blue. This is not a surprise, as the primary regions are broken up into representations of individual body parts and the higher-order regions are not. The model captured this trend through target region coordinate systems where some of the defining points landed far outside the region (Supplementary Figure 3 a, columns "model").

Conversely, we made no assumption of complete coverage of the target region. While it was largely complete in the upstream pathways of the visual system (Fig. 3), according to the data this was not the case for feedforward projections in the somatomotor system, and the model captured this trend (Supplementary Figure 3b). This is in line with experimental findings in rat [5] and mouse [6]. Regions as targets of feedforward projections were only incompletely covered, and in fact the same parts of the regions were engaged in both feedforward and feedback projections with a given primary somatosensory region. Furthermore, as a group, the primary somatosensory regions engage all parts of other regions about equally. For example the parts engaged by SSp-ll and SSp-m are non-overlapping and together completely cover MOp and SSs (Supplementary Figure 3b, left vs. right).

Overall, mapping coverage was complete for 50% of projections on the source side and for 62% of projections on the target side. Over the whole neocortex, average source region coverage was 89% and target region coverage was 84% (Supplementary Figure 3 c, d). Incomplete target region coverage was taken into account in the scaling of anatomical projection strengths.

2 Supplementary Discussion

2.1 Assumptions made during modelling

Supplementary Table 2 summarizes the assumptions made to formulate the model. Data assumptions are about the nature of the biological data used and are made to interpolate missing data points. As more and more data becomes available, fewer data assumption will be required. For example, we had to assume symmetry to build a full neocortical model because the chosen biological dataset was focused on the right hemisphere. With the addition of more data points in the left hemisphere, the assumption could be abolished and potential asymmetries revealed. Indeed, there is evidence of lateral asymmetries, also in non-human animals[7], although it is unclear to what degree they are population-level tendencies that can be captured by a model based on data pooled from many individuals.

Structuring assumptions were made to structure the data, and reduce its complexity or to break up the problem to reduce its scale. For example, the clear split between local and global connectivity allowed us to tackle the individual sub-problems separately. These are shortcuts that are not inherently required and could potentially be phased out in future, improved versions of this model. The first candidate for such a future improvement is derivation of layer profiles of projections. The current approach proved challenging, due to the great variability in the biological data as reported by the voxelized connectome model, and consequently remains imperfect. A supplementary approach would be to analyze individual whole-brain axons, similar to our approach to region targeting. However, this comes with the caveat that analyzing individual axons in the context of an average brain parcellation scheme - such as the Allen common coordinate framework - leads to inaccuracies. While this is unlikely to upset the results for region targeting, the more fine-grained layer targeting will be more affected. Finally, for a complete model, axons originating from many different brain regions and layers would be required. Ultimately, whole-brain electron microscopy holds promise to solve these issues.

Modelling assumptions are integral to the model and relate to hypotheses about the biological system. We consider an assumption generalizing if it has been validated for part of the neocortex and is used for all of it. We also inherit all assumptions that were made in order to generate the voxelized connectivity model that we used.

Finally, there is the implicit assumption of completeness, that our model captures all pertinent biological principles. Such an assumption is made in all modelling work.

2.2 A potential future of genetically encoded p-types

We implemented the targeting of individual brain regions with a tree-based model that conceptualizes the axon growing throughout the brain. While we do not make any claims about an anatomical basis of this conceptual model at this point, it may prove fruitful to relate it to anatomical fiber tracts in the future. It is possible that the fiber tracts implement a super-graph that the trees we identified for individual projection types are subgraphs of. More specific projection classes may exist with a molecular encoding of which edges of the super-graph are present or absent. Indeed, genetic signatures of individual neurons have already been systematically analyzed and linked to axonal projections[8,9]. In the future the coarse-grained projection types used here may be replaced by genetically defined types identified in these works, including their region-specificity.

3 Supplementary tables

Prefrontal	FRP	Frontal pole, cerebral cortex
	MOs	Secondary motor area
	ACAd	Anterior cingulate area, dorsal part
	ACAv	Anterior cingulate area, ventral part
	PL	Prelimbic area
	ILA	Infralimbic area
	ORBl	Orbital area, lateral part
	ORBm	Orbital area, medial part
	ORBvl	Orbital area, ventrolateral part
Anterolateral	AId	Agranular insular area, dorsal part
	AIv	Agranular insular area, ventral part
	AIp	Agranular insular area, posterior part
	GU	Gustatory areas
	VISC	Visceral area
Somatomotor	SSs	Supplemental somatosensory area
	SSp-bfd	Primary somatosensory area, barrel field
	SSp-tr	Primary somatosensory area, trunk
	SSp-ll	Primary somatosensory area, lower limb
	SSp-ul	Primary somatosensory area, upper limb
	SSp-un	Primary somatosensory area, unassigned
	SSp-n	Primary somatosensory area, nose
	SSp-m	Primary somatosensory area, mouth
	MOp	Primary motor area
Visual	VISal	Anterolateral visual area
	VISl	Lateral visual area
	VISp	Primary visual area
	VISpl	Posterolateral visual area
	VISli	Laterointermediate area
	VISpor	Postrhinal area
	VISrl	Rostrolateral visual area
Medial	VISa	Anterior area
	VISam	Anteromedial visual area
	VISpm	posteromedial visual area
	RSPagl	Retrosplenial area, lateral agranular part
	RSPd	Retrosplenial area, dorsal part
	RSPv	Retrosplenial area, ventral part
Temporal	AUDd	Dorsal auditory area
	AUDp	Primary auditory area
	AUDpo	Posterior auditory area
	AUDv	Ventral auditory area
	TEa	Temporal association areas
	PERI	Perirhinal area
	ECT	Ectorhinal area

Supplementary Table 1: Order of brain modules (left) and regions (middle and right) used throughout the manuscript

4 Supplementary figures

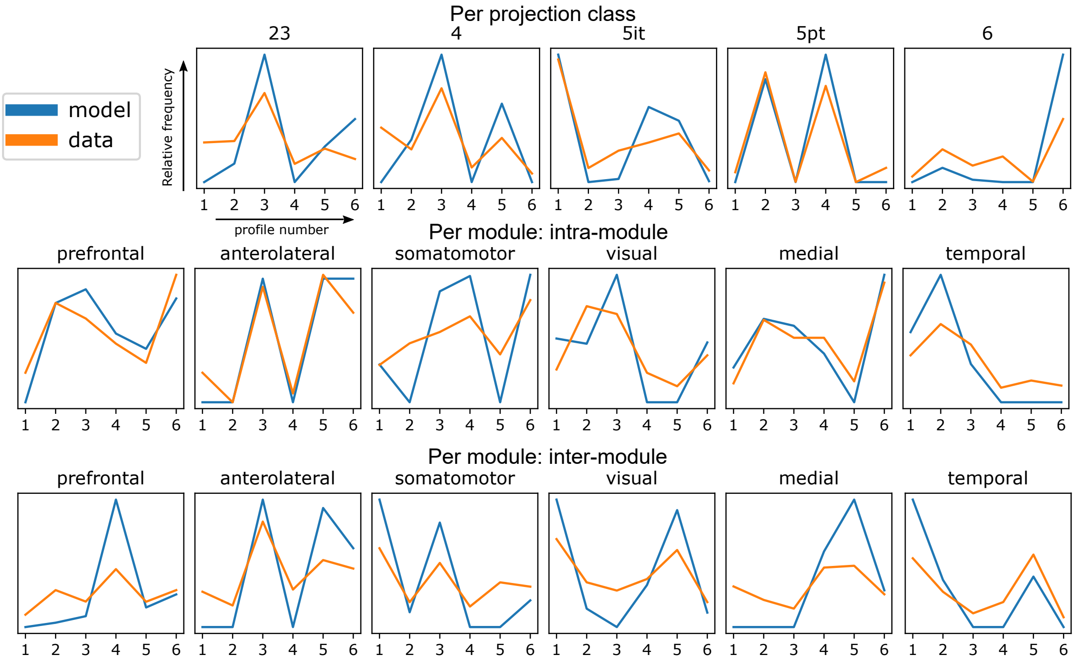

Supplementary Figure 1: **Validating layer profile frequencies.** Comparing resulting layer profile frequencies for projection classes and modules to the data of Harris et al. [2]

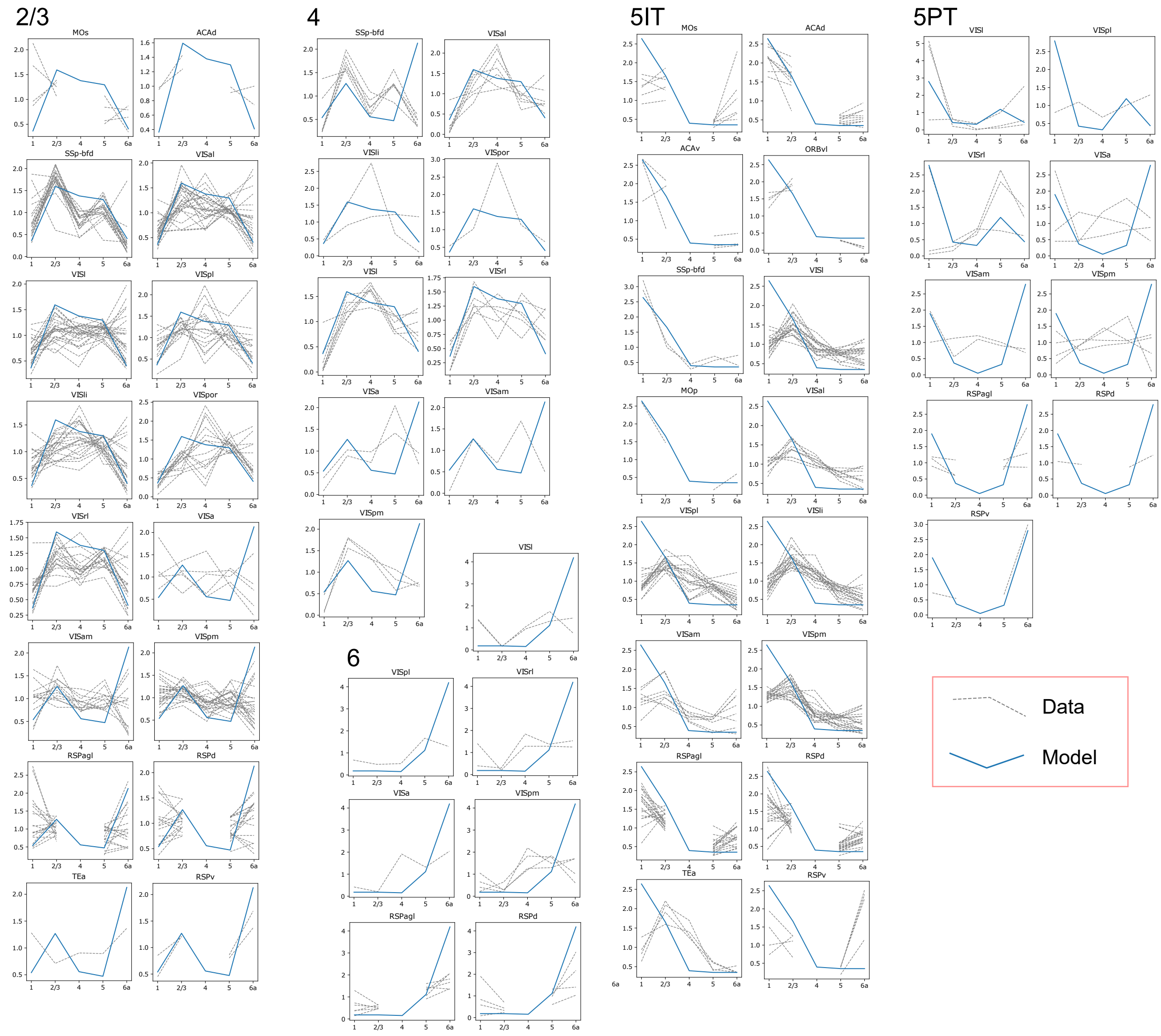

Supplementary Figure 2: **Validating predictions against raw layer profiles.** Comparing raw layer profiles from individual experiments in the voxelized projection model (grey, Knox et al.[1]) to our model prediction in all regions due to projections from neurons in MOs. Only data where the projection strength in a region is more than 1% of the total strength in all regions is shown.

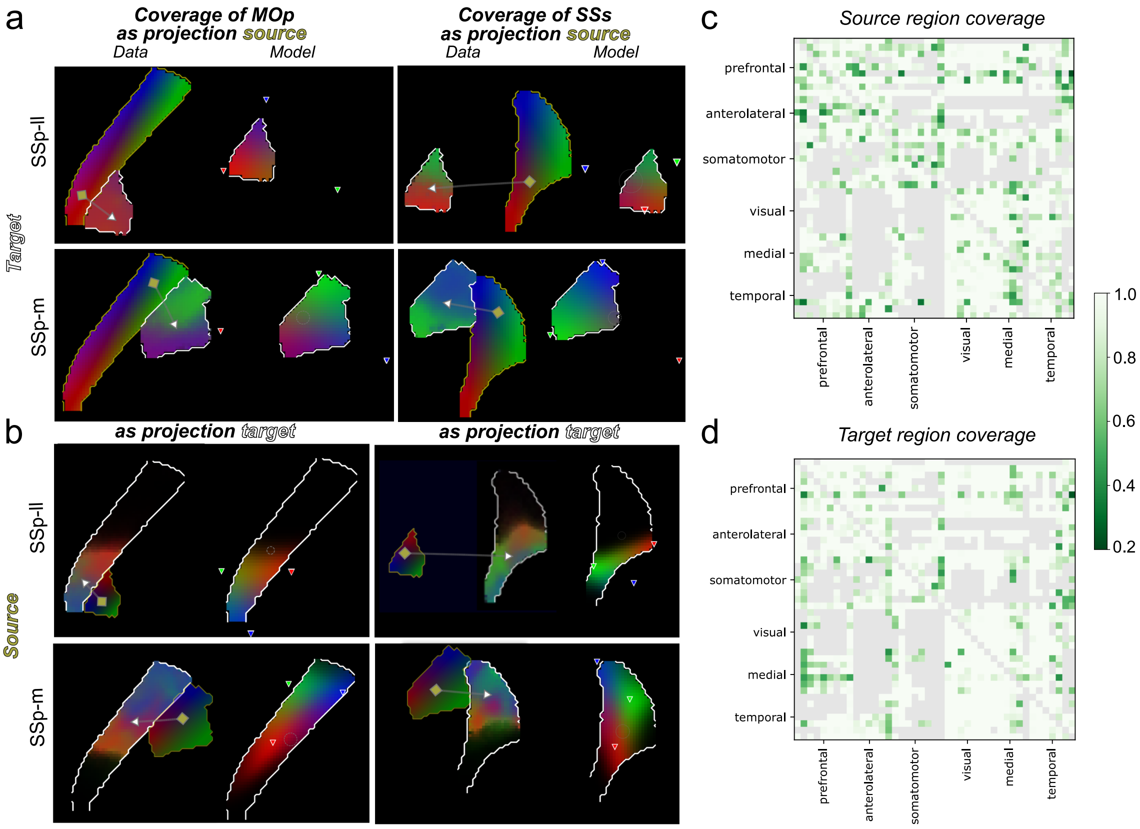

Supplementary Figure 3: **Incomplete mapping.** **a**, Exemplary feedback projections in the somatomotor module only incompletely cover the source regions. Projections are MOp to SSp-ll, SSs to SSp-ll, MOp to SSp-m and MOs to SSp-m (top left to bottom right). Relative projection strengths (left) and the resulting barycentric model (right), including the defining points (colored triangles) are depicted. **b**, Exemplary feedforward projections in the somatomotor module only incompletely cover the target regions. As in **a** but source and target region are swapped (SSp-ll to MOp, SSp-ll to SSs, SSp-m to MOp, SSp-m to SSs). **c**, Fractions of the source regions covered by ipsi-lateral projections. Grey areas depict projections weaker than a threshold value or connectivity within a region. Ordering as in Fig. 4 and Supplementary Table 1. **d**, As **c**, but coverage of the target region.

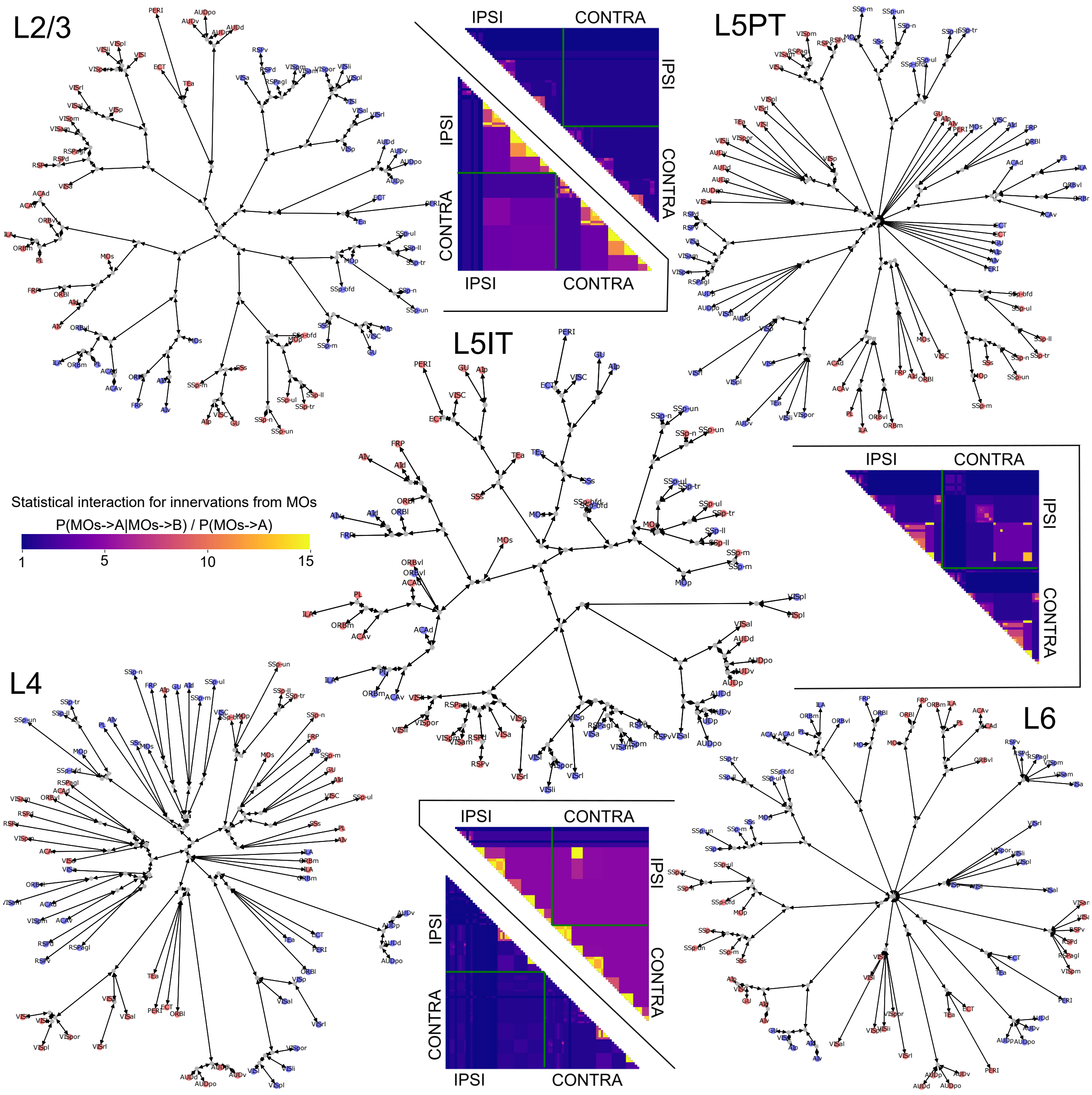

Supplementary Figure 4: **The final tree models for all projection classes.** Edge lengths depicted are the mean of lengths in either direction. Blue / red nodes are leaves representing brain regions in either hemisphere. Depicted alongside each tree are the predicted statistical interactions between innervations of ipsi- and contra-lateral regions from MOs as in Fig. 5d.

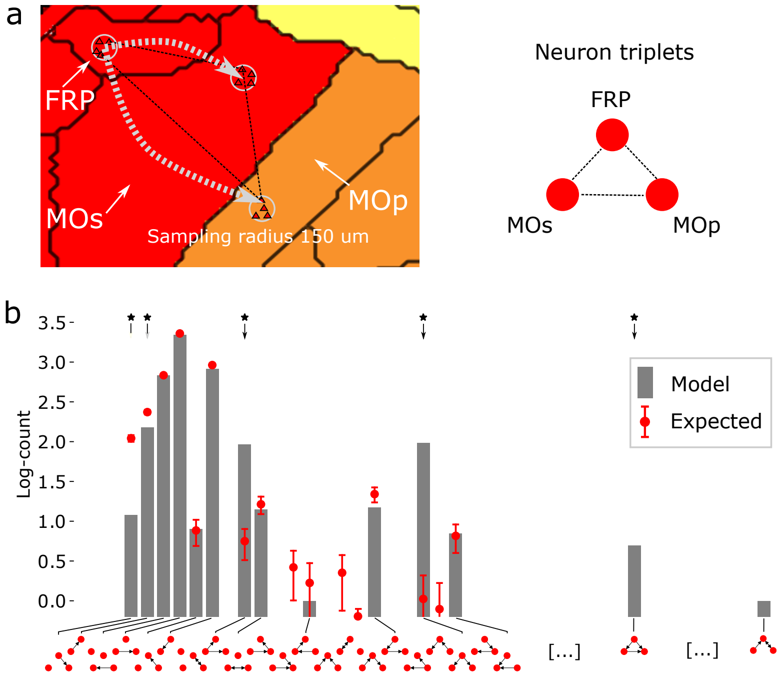

Supplementary Figure 5: **Motifs of neuron triplets.** Motifs where one neuron is in MOs, one in MOp and one in FRP. **a**. Connectivity between individual neurons in the three regions was sampled by defining a 150μm subvolume in FRP, then finding the centers of the projection from the subvolume to MOs and MOp according to the mapping (dashed arrows) and defining subvolumes with the same radius around them. **b**. We randomly picked 100,000 triplets of of neurons in the three subvolumes and counted the counts of unique motifs emerging (grey bars) and compared them to the numbers expected from the unidirectional connection probabilities between the subvolumes (red dots and bars; mean and std; binomial model). Asterisks and arrows indicate statistically significant difference ($p < 0.01$; corrected for multiple comparisons).

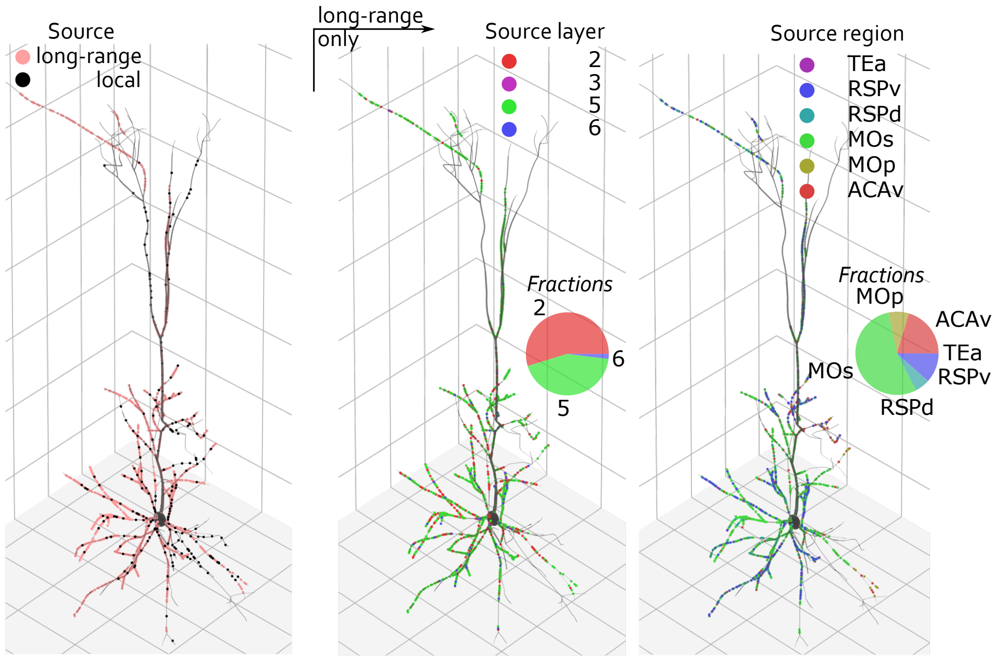

Supplementary Figure 6: Modeled synaptic innervation of an exemplary PC in layer 5 of ACAd in terms of the origin of each synaptic contact. Local vs. long-range (left), by source layer (middle), or by source region (right).

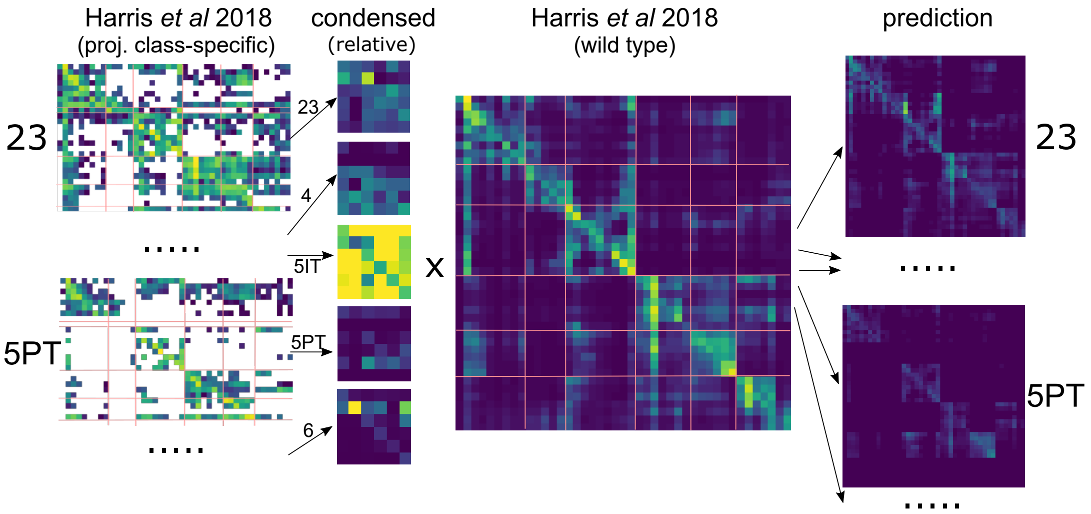

Supplementary Figure 7: **Prediction of 43 by 43 projection class-specific connection matrices**

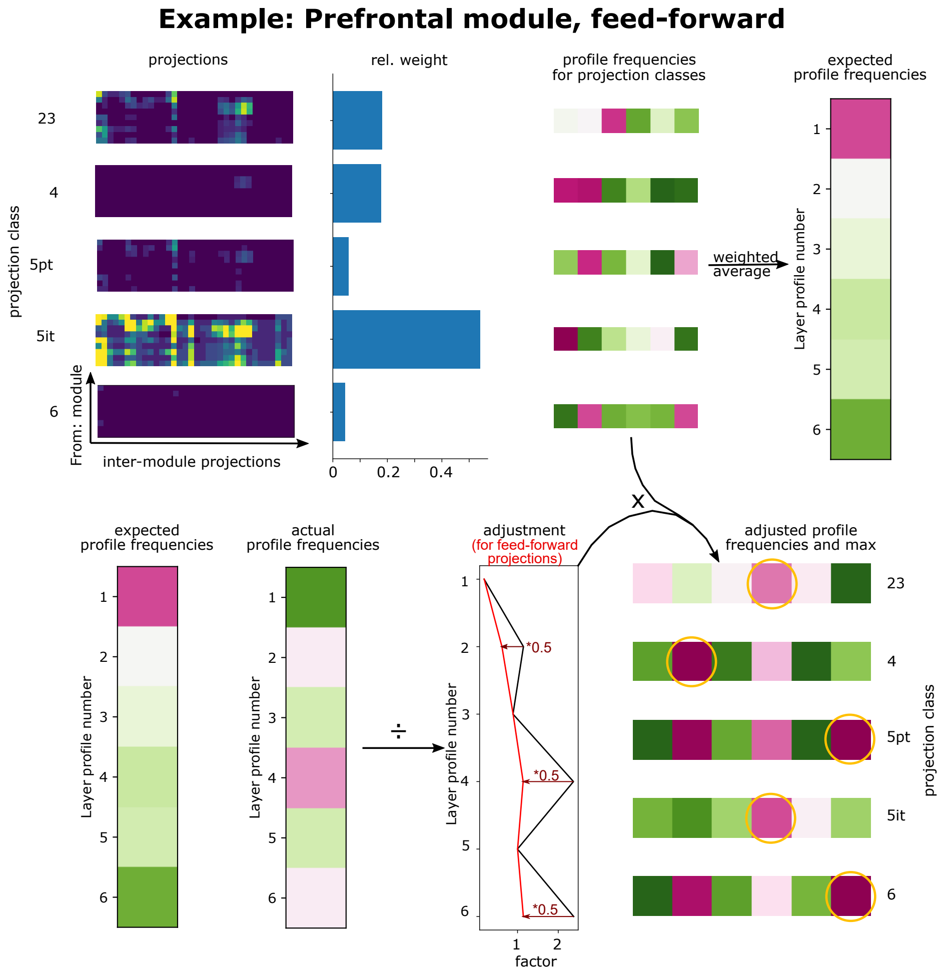

Supplementary Figure 8: **Predicting layer profile frequencies.** Predictions for feed-forward inter-module connections from the prefrontal module and all projection classes

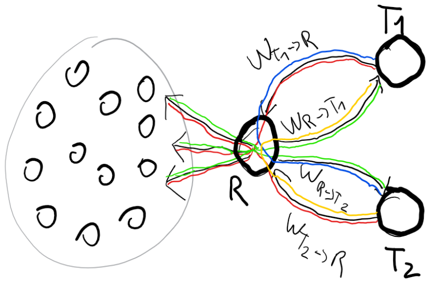

$w_{T_1 \to R} - w_{T_2 \to R} \approx |M[T_1, :]| - |M[T_2, :]|$

$w_{R \to T_1} - w_{R \to T_2} \approx |M[:, T_1]| - |M[:, T_2]|$

$M[T_1, T_2] = w_{T_1 \to R} + w_{R \to T_2}$

$M[T_2, T_1] = w_{T_2 \to R} + w_{R \to T_1}$

Supplementary Figure 9: **P-type model construction method.** The weights in the p-type model tree can be estimated for triad motifs of two siblings and their parent from the matrix of path lengths between leaf nodes and four known shortest paths in the motif. Blue: shortest paths from leaf nodes to all other leaves; red: shortest paths from all other leaves to the leaf nodes; green and orange: shortest paths between the leaf nodes.

Bibliography

[1] Joseph E. Knox, Kameron Decker Harris, Nile Graddis, Jennifer D. Whitesell, Hongkui Zeng, Julie A. Harris, Eric Shea-Brown, and Stefan Mihalas. High resolution data-driven model of the mouse connectome. April 2018. doi: 10.1101/293019. URL `http://biorxiv.org/lookup/doi/10.1101/293019`.

[2] Julie A Harris, Stefan Mihalas, Karla E Hirokawa, Jennifer D Whitesell, Joseph Knox, Amy Bernard, Phillip Bohn, Shiella Caldejon, Linzy Casal, Andrew Cho, David Feng, Nathalie Gaudreault, Charles Gerfen, Nile Graddis, Peter A Groblewski, Alex Henry, Anh Ho, Robert Howard, Leonard Kuan, Jerome Lecoq, Jennifer Luviano, Stephen McConoghy, Marty Mortrud, Maitham Naeemi, Lydia Ng, Seung W Oh, Benjamin Ouellette, Staci Sorensen, Wayne Wakeman, Quanxin Wang, Ali Williford, John Phillips, Allan Jones, Christof Koch, and Hongkui Zeng. The organization of intracortical connections by layer and cell class in the mouse brain. April 2018. doi: 10.1101/292961. URL `http://biorxiv.org/lookup/doi/10.1101/292961`.

[3] Michael W. Reimann, James G. King, Eilif B. Muller, Srikanth Ramaswamy, and Henry Markram. An algorithm to predict the connectome of neural microcircuits. *Frontiers in Computational Neuroscience*, 9, October 2015. doi: 10.3389/fncom.2015.00120.

[4] Henry Markram, Eilif Muller, Srikanth Ramaswamy, Michael W. Reimann, Marwan Abdellah, Carlos Aguado Sanchez, Anastasia Ailamaki, Lidia Alonso-Nanclares, Nicolas Antille, Selim Arsever, Guy Antoine Atenekeng Kahou, Thomas K. Berger, Ahmet Bilgili, Nenad Buncic, Athanassia Chalimourda, Giuseppe Chindemi, Jean-Denis Courcol, Fabien Delalondre, Vincent Delattre, Shaul Druckmann, Raphael Dumusc, James Dynes, Stefan Eilemann, Eyal Gal, Michael Emiel Gevaert, Jean-Pierre Ghobril, Albert Gidon, Joe W. Graham, Anirudh Gupta, Valentin Haenel, Etay Hay, Thomas Heinis, Juan B. Hernando, Michael Hines, Lida Kanari, Daniel Keller, John Kenyon, Georges Khazen, Yihwa Kim, James G. King, Zoltan Kisvarday, Pramod Kumbhar, Sébastien Lasserre, Jean-Vincent Le Bé, Bruno R.C. Magalhães, Angel Merchán-Pérez, Julie Meystre, Benjamin Roy Morrice, Jeffrey Muller, Alberto Muñoz-Céspedes, Shruti Muralidhar, Keerthan Muthurasa, Daniel Nachbaur, Taylor H. Newton, Max Nolte, Aleksandr Ovcharenko, Juan Palacios, Luis Pastor, Rodrigo Perin, Rajnish Ranjan, Imad Riachi, José-Rodrigo Rodríguez, Juan Luis Riquelme, Christian Rössert, Konstantinos Sfyrakis, Ying Shi, Julian C. Shillcock, Gilad Silberberg, Ricardo Silva, Farhan Tauheed, Martin Telefont, Maria Toledo-Rodriguez, Thomas Tränkler, Werner Van Geit, Jafet Villafranca Díaz, Richard Walker, Yun Wang, Stefano M. Zaninetta, Javier DeFelipe, Sean L. Hill, Idan Segev, and Felix Schürmann. Reconstruction and Simulation of Neocortical Microcircuitry. *Cell*, 163(2):456–492, October 2015. doi: 10.1016/j.cell.2015.09.029.

[5] Jared B. Smith and Kevin D. Alloway. Rat whisker motor cortex is subdivided into sensory-input and motor-output areas. *Frontiers in Neural Circuits*, 7, 2013. doi: 10.3389/fncir.2013.00004.

[6] B. A. Suter and G. M. G. Shepherd. Reciprocal Interareal Connections to Corticospinal Neurons in Mouse M1 and S2. *Journal of Neuroscience*, 35(7):2959–2974, February 2015. doi: 10.1523/JNEUROSCI.4287-14.2015.

[7] Michael C Corballis. The evolution and genetics of cerebral asymmetry. *Philosophical Transactions of the Royal Society B: Biological Sciences*, 364(1519):867–879, April 2009. doi: 10.1098/rstb.2008.0232.

[8] Bosiljka Tasic, Vilas Menon, Thuc Nghi Nguyen, Tae Kyung Kim, Tim Jarsky, Zizhen Yao, Boaz Levi, Lucas T Gray, Staci A Sorensen, Tim Dolbeare, Darren Bertagnolli, Jeff Goldy, Nadiya Shapovalova, Sheana Parry, Changkyu Lee, Kimberly Smith, Amy Bernard, Linda Madisen, Susan M Sunkin, Michael Hawrylycz, Christof Koch, and Hongkui Zeng. Adult mouse cortical cell taxonomy revealed by single cell transcriptomics. *Nature Neuroscience*, 19(2):335–346, February 2016. doi: 10.1038/nn.4216.

[9] Bosiljka Tasic, Zizhen Yao, Lucas T. Graybuck, Kimberly A. Smith, Thuc Nghi Nguyen, Darren Bertagnolli, Jeff Goldy, Emma Garren, Michael N. Economo, Sarada Viswanathan, Osnat Penn, Trygve Bakken, Vilas Menon, Jeremy Miller, Olivia Fong, Karla E. Hirokawa, Kanan Lathia, Christine Rimorin, Michael Tieu, Rachael Larsen, Tamara Casper, Eliza Barkan, Matthew Kroll, Sheana Parry, Nadiya V. Shapovalova, Daniel Hirschstein, Julie Pendergraft, Heather A. Sullivan, Tae Kyung Kim, Aaron Szafer, Nick Dee, Peter Groblewski, Ian Wickersham, Ali Cetin, Julie A. Harris, Boaz P. Levi, Susan M. Sunkin, Linda Madisen, Tanya L. Daigle, Loren Looger, Amy Bernard, John Phillips, Ed Lein, Michael Hawrylycz, Karel Svoboda, Allan R. Jones, Christof Koch, and Hongkui Zeng. Shared and distinct transcriptomic cell types across neocortical areas. *Nature*, 563(7729):72–78, November 2018. doi: 10.1038/s41586-018-0654-5.
